# The angiotensin-converting enzyme 2 (ACE2) receptor in the prevention and treatment of COVID-19 are distinctly different paradigms

**DOI:** 10.1186/s40885-020-00147-x

**Published:** 2020-07-15

**Authors:** Craig Steven McLachlan

**Affiliations:** Torrens University Australia, Health Vertical, 5/235 Pyrmont St, Pyrmont, NSW 2009 Australia

**Keywords:** ACE2 receptor, COVID-19, SIRT1, ACE inhibitors, ARBs, Vitamin C, Metformin, Resveratrol, Coronavirus, SAR-CoV2

## Abstract

There is current debate concerning the use of angiotensin-converting enzyme (ACE) inhibitors or angiotensin II type 1 receptor blockers (ARBs), for hypertension management, during COVID-19 infection. Specifically, the suggestion has been made that ACE inhibitors or ARBs could theoretically contribute to infection via increasing ACE2 receptor expression and hence increase viral load. The ACE2 receptor is responsible for binding the SAR-CoV2 viral spike and causing COVID-19 infection. What makes the argument somewhat obtuse for ACE inhibitors or ARBs is that ACE2 receptor expression can be increased by compounds that activate or increase the expression of SIRT1. Henceforth common dietary interventions, vitamins and nutrients may directly or indirectly influence the cellular expression of the ACE2 receptor. There are many common compounds that can increase the expression of the ACE2 receptor including Vitamin C, Metformin, Resveratrol, Vitamin B3 and Vitamin D. It is important to acknowledge that down-regulation or blocking the cellular ACE2 receptor will likely be pro-inflammatory and may contribute to end organ pathology and mortality in COVID-19. In conclusion from the perspective of the ACE2 receptor, COVID-19 prevention and treatment are distinctly different. This letter reflects on this current debate and suggests angiotensin-converting enzyme inhibitors and ARBs are likely beneficial during COVID-19 infection for hypertensive and normotensive patients.

## Main text

The angiotensin-converting enzyme 2 (ACE2) receptor acts as the receptor-binding domain for the SAR-CoV2 virus spike complex [1]. This permits viral attachment, fusion and intracellular entry and infection with COVID-19 [1]. Compounds that may increase the expression of the ACE2 receptor have received media interest [2]. Particularly, from the point of view, that increased expression of the ACE2 receptor, may make SAR-CoV2 more infective via increasing viral load, morbidity and mortality [2–4]. From the perspective of the ACE2 receptor, COVID-19 prevention and treatment are distinctly different.

COVID-19 prevention via targeting the cellular ACE2 receptor is theoretically interesting, it is however, not a practically useful strategy and could potentially increase mortality [2, 3]. On the other hand, there has been interest in delivering soluble ACE2 receptors that may bind SAR-CoV2 spikes and deactivate the virus. This would be a practical solution, as this leaves the cellular ACE2 receptor system intact (if all virus is bound to decoy soluble ACE2 receptors) [5]. Soluble ACE2 receptors can reduce vial load up to 5000-fold (in cell culture) and be used to reduce viral load early or as a treatment option to preserve lung during acute and severe respiratory involvement [6].

Some investigator groups have suggested that ACE inhibitors may be a logical choice for all patients with COVID-19 infection [7]. This is irrespective if patients have pre-existing hypertension or not. The rationale for giving ACE inhibitors to all patients is that it may enhance the expression of ACE2 receptors that further reduce cellular inflammation [2, 7]. There is limited evidence that ACE inhibitors or angiotensin II type 1 receptor blockers (ARB’s) may up-regulate ACE2 receptor mRNA and or expression [2].

Importantly, the COVID-19 infection effectively down-regulates the ACE2 receptor via attaching to infection-related transcription factors at the ACE2 regulatory regions [2]. ANG II also reduces the expression of the ACE2 receptor. Cardiac, lung, liver and renal damage is responsible for morbidity and mortality in COVID-19 [8]. These organ systems have a higher expression of ACE2 receptors than other bodily systems. Because ACE2 converts Ang II to Ang [1–7], down-regulation of the ACE2 receptor would leave critical organ systems susceptible to hyper-inflammation via unopposed increases in Angiotensin II (ANG II) [2]. Respiratory distress in COVID-19 and SARS is likely associated with reduced ACE2 receptor expression [2, 7–9]. Individuals with diabetes, high blood pressure, metabolic syndrome and advanced age (with reduced organ ACE2 expression) are prone to higher fatality rates [10].

There has also been significant media discussion around blood pressure management in COVID-19 [2]. Blood pressure therapeutics that target the angiotensin system have been discussed at length in journal editorial articles. Professional societies have now released position statements stating that ACE inhibitors and ARBs should not be withdrawn either as a preventive measure or as a treatment option in COVID-19 [2]. Angiotensin II formation would prevail if ACE inhibitors or ARBs as a mono-class are withdrawn in hypertensive patients [2, 4]. In hypertension ACE inhibitors are serving to restore the balance of ACE2 to ANG II formation. It is possible that ACE inhibitors may reduce the down regulation of ACE2 receptors via reducing ANG II formation in COVID-19 [2, 7, 9].

Interestingly, it is not only ACE inhibitors and ARBs at therapeutic doses that can influence ACE2 expression. There are other mediators that may increase the expression of ACE2 such as increased potassium intake [11], intermittent fasting [12], nicotine [13] and Vitamin D [14].

It is important to consider that ACE2 expression can be increased by compounds that activate or increase the expression of SIRT1 [15, 16]. SIRT1 is expressed next to the promotor region of ACE2 gene hence increased expression and or enhanced functional activation of SIRT1 is associated with an increase in expression of ACE2 [16]. Common mediators that interact positively with SIRT1 expression or activation (directly or indirectly) are calorie restriction [17], resveratrol [18, 19], Vitamin C [20, 21], aspirin [20], metformin [22], vitamin B3 [23]. This may provide a mechanistic explanation as to why high dose Vitamin C is a potential rescue therapy for severe acute respiratory distress syndrome in COVID-19 [10].

Key points with respect to COVID-19 respiratory infection

- Down-regulation or blocking the cellular ACE2 receptor will be pro-inflammatory and may contribute to end organ pathology.
- Therapeutics that stimulate the functional expression of ACE2 receptor or inhibit ACE II could be a useful therapeutic approach.
- ACE inhibitors or ARBs may be safe in both hypertensive and normotensive patients
- Editorial discussion whether ACE inhibitors or ARBs should be discontinued or continued with respect to ACE2 expression, seems obtuse. Particularly when other therapeutics, dietary interventions, vitamins and nutrients may directly or indirectly may influence the cellular expression of the ACE2 receptor.
- There are many common compounds that can increase the expression of the ACE2 receptor including Vitamin C, Metformin, Resveratrol, Vitamin B3 and Vitamin D.

## Data Availability

Not applicable.

## Abbreviations

ANG II: Angiotensin II
ACE2: Angiotensin-converting enzyme 2
ARB’s: Angiotensin II type 1 receptor blockers
mRNA: Messenger RNA
SARS-CoV-2: Severe acute respiratory syndrome coronavirus 2
SARS: Severe acute respiratory syndrome
SIRT1: Sirtuin 1
